# Policy foundations for transformation: a gender analysis of adolescent health policy documents in South Africa

**DOI:** 10.1093/heapol/czab041

**Published:** 2021-04-14

**Authors:** Tanya Jacobs, Asha George, Michelle De Jong

**Affiliations:** School of Public Health, University of the Western Cape, Bellville, Robert Sobukwe Rd, Western Cape 7535, South Africa; School of Public Health, University of the Western Cape, Bellville, Robert Sobukwe Rd, Western Cape 7535, South Africa; School of Public Health, University of the Western Cape, Bellville, Robert Sobukwe Rd, Western Cape 7535, South Africa

**Keywords:** Gender analysis, gender, adolescent health, policy analysis, critical discourse analysis, intersectionality

## Abstract

The Sustainable Development Goals (SDGs) and the United Nations Global Strategy (2016–30) emphasize that all women, children and adolescents ‘survive, thrive and transform’. A key element of this global policy framework is that gender equality is a stand-alone goal as well as a cross-cutting priority. Gender inequality and intersecting social and structural determinants shape health systems, including the content of policy documents, with implications for implementation. This article applies a gender lens to policy documents by national government bodies that have mandates on adolescent health in South Africa. Data were 15 policy documents, authored between 2003 and 2018, by multiple actors. The content analysis was guided by key lines of enquiry, and policy documents were classified along the continuum of gender blind to gender transformative. Only three policy documents defined gender, and if gender was addressed, it was mostly in gender-sensitive ways, at times gender specific, but rarely gender transformative. Building on this, a critical discourse analysis identified what is problematized and what is left unproblematized by actors, identifying the key interrelated dominant and marginalized discourses, as well as the ‘silences’ embedded in policy documents. The discourse analysis revealed that dominant and marginalized discourses reflect how gender is conceptualized as fixed, categorical identities, vs as fluid social processes, with implications for how rights and risks are understood. The discourses substantiate an over-riding focus on adolescent girls, outside of the context of power relations, with minimal attention to boys in terms of their own health or through a gender lens, as well as little consideration of LGBTIQ+ adolescents beyond HIV. Dynamic and complex relationships exist between the South Africa context, actors, content and processes, in shaping both how gender is problematized and how ‘solutions’ are represented in these policies. How gender is conceptualized matters, both for policy analysis and for praxis, and policy documents can be part of foundations for transforming gender and intersecting power relations.

**Key messages**

- Integrating gender, both as theoretical and as methodological approach, is an important contribution to Health Policy Analysis (HPA), with implications both for analysis of policy and for implementation.
- Policy documents are socially constructed, and the content represents ideas, understandings and assumptions about gender in the South African context, and by actors, shaping both what is problematized and what is unproblematized, i.e. left out.
- Our analysis describes a complex landscape consisting of multiple actors and foci that lack coherence and alignment as well lack of detailed gender analyses. In addition, our gender analysis identified key interrelated, often juxtaposed, dominant and marginalized discourses, as well as the ‘silences’, embedded in the policy documents, with significant implications for policy implementation.
- Researchers, policymakers and implementers should integrate gender-transformative approaches as part of addressing gender inequality as a key social and structural determinant of adolescent health, and policy documents are foundations to part of re-imagining policy and health systems.

## Introduction

The Sustainable Development Goals (SDGs) adopted by the United Nations (United Nations General Assembly, 2015) are aligned with the United Nations Secretary General’s Global Strategy (2016−30). Both call for all women, children and adolescents to ‘survive, thrive and transform’ as a priority for global health. This goes beyond ensuring that women and children ‘survive’ against threats to mortality addressed by healthcare interventions and represents a broader vision of health and well-being, i.e. ‘thrive,’ while also addressing development more holistically, i.e. ‘transform’. Within this reconceptualization, gender equality is both a stand-alone goal and a cross-cutting concern, and adolescents are a key priority.

In South Africa, social transformation inclusive of gender equality and adolescent rights is also a national priority for government and civil society (South African National AIDS Council, 2017; Toska *et al.*, 2019). However, despite the Constitution of the Republic of South Africa of 1996 being grounded in equality, significant challenges and intersecting inequalities remain post-apartheid. For example, gender inequality contributes to the extremely high prevalence of gender-based violence and HIV incidence in adolescent girls and young women in particular (Government of South Africa, 2020; Nduna, 2020).

Gender as a social construct is fluid and relational and refers to the roles, attributes, norms and behaviours considered to be appropriate for girls/women, boys/men and other genders (World Health Organization, 2020; Springer *et al.*, 2012; Connell, 2012). Gender inequality also intersects with various other axes of inequality in terms of race, class, sexual orientation and (dis)ability, thus compounding power dynamics and stratification (Hankivsky, 2014; Larson *et al.*, 2016; Morgan *et al.*, 2016).

Some ways in which gender inequalities are generated or contested include how health policy is shaped by gender, in both its content and its processes (Bacchi and Eveline, 2010; Lombardo *et al.*, 2017). The gendered power relationships and assumptions that are often embedded within the content of policy documents are not always immediately obvious or explicit. This is problematic because, unacknowledged, these kinds of assumptions in policy documents can function to reproduce the status quo and block the transformation of gender inequality.

Health policy analysis (HPA) aims to make visible power relations within policy processes and to integrate this into the study of health policies and systems (Gilson and Raphaely, 2008; Gilson *et al.*, 2018). The intentions, ideological positions, values and meaning making of/by actors are central to the construction of policy (McDougall, 2016; Gilson *et al.*, 2018), and there is a paucity of literature in HPA that foregrounds this in terms of gendered power relations.

To better understand the dynamic interaction between the South African context, policy actors and content, as part of policy processes, this paper applies a critical gender lens to the content of national government health policy documents relevant to adolescent health in South Africa. Furthermore, the paper explores the nexus of the construction of gender and related discourses in adolescent health and raises considerations for what this means for policy analysis and praxis in South Africa.

## Methodology

### Theoretical and methodological approach

The analysis of socially constituted and contested policy processes is the focus of a growing body of literature in HPA i.e. rooted in post-structuralism that recognizes that all social phenomena, including policies, are socially constructed (Shiffman *et al.*, 2004; Ingram *et al.*, 2007). An examination of policy documents as ‘artefacts’ is one way of understanding the underlying meanings and consequences of how policies are socially constructed by policy actors and how these intersect with local social, political, economic, and cultural contexts, (Parkhurst *et al.*, 2015) including the policy discourses involved.

Discourse analysis, an element of HPA, seeks to further understand meanings underlying policies by examining how representations of ‘problems’ come about, structuring ‘solutions’ and subjectivities (Bacchi and Eveline, 2010; Bacchi, 2016). While the ways in which ‘problems’ are commonly conceptualized in public health are not always perceived as socially constructed, the policies and the problems they seek to address are not neutral. Furthermore, the consequences of constructing certain issues as ‘problems’ are often unacknowledged (Walt *et al.*, 2008; Bacchi, 2016; Gilson *et al.*, 2018)

Critical discourse analysis is a growing theoretical and methodological approach in the analysis of health policy. It is informed by critical social theories and draws its theoretical antecedents from the body of work by Foucault (Fairclough, 2013; Wodak and Meyer, 2016). It can be used in combination with any other methodology and is ‘critical’ in that it aims to illustrate the non-obvious ways in which language or texts are intricately linked to power relations and ideological positions.

Despite the increased interest and utilization of discourse analysis in HPA (Harmer, 2011; Raphael, 2011; Shapiro, 2014; Parkhurst *et al.*, 2015; Evans-Agnew *et al.*, 2016), there is a paucity of discourse analysis with a gender lens, let alone any applied to adolescent health policy. While there is an increasing focus on adolescent health in South Africa (Cluver *et al.*, 2019; Toska *et al.*, 2019), to our knowledge our research using a gender analysis of adolescent health policy documents in South Africa is unique, both in terms of focus and methodology.

When working with the notion of discourse as ‘social practice’ and policies as productive, it is evident that discourses are created in the dialectical and dynamic relationships between content of policy documents and social contexts (Bacchi, 2010; Fairclough, 2013; Wodak and Meyer, 2016). Discourse analysis allows us to make interpretative connections and complex relationships between the content of policies, actors and social contexts. By applying a gender analysis, we are able to focus on gender discourses in national government’s adolescent health policy documents, in order to identify discourses which sustain, contest and perpetuate complex patriarchal power relations, in order to transform them (Lazar, 2007).

### Data collection

Given our focus on policy documents by national government bodies that have the mandate and are technically responsible for policy development related to adolescent health in South Africa, we iteratively searched for them on national government websites and the internet with a focus on the previous 15 years, i.e. from 2003 to 2018. Search terms included a combination of ‘youth’, ‘young’, ‘adolescent’, ‘health’, ‘gender’, ‘multi-sectoral’, ‘school health’, ‘sexual and reproductive health’, ‘mental health’, ‘policy’ and ‘policy development’. Key experts in the field were also consulted to ensure that the search for policy documents was comprehensive. The scope and relevance of the national policy documents related to their direct contribution to adolescent health were collected and reviewed by the first and second authors.

### Data analysis

Policy documents were uploaded into ATLAS.ti and a coding framework, and code list were developed using an initial broad coding structure derived from propositions in the research. The initial coding framework was applied to four policy documents by the first author and reviewed by the second author before completion of coding processes. A deductive analytical approach was initially used, whereby the data were organized using the pre-defined codes which included gender, adolescent, youth, health, inequality, rights, participation and so on (Bowen, 2009). These were linked to the key lines of enquiry and an allowance was made for new themes to emerge in the analysis process. These additional codes included sexual orientation and gender identity, non-binary, determinant and responsive, as examples.

For the content analysis, key lines of enquiry included the following questions in terms of the content of the adolescent health policies, as a basis for understanding policy discourses:

- How is adolescence constructed?
- How is adolescent health constructed?
- How is gender constructed?
- How is gender inequality and its intersectionality with other form of inequality constructed?
- How are adolescent rights and engagement constructed?

Further, for the content analysis shown in Table S1, the following types of questions were asked in relation to gender: how gender and key gender terms are defined, the extent and nature of gender analysis undertaken, and whether gender inequality is also recognized as a social and structural determinant of health. In addition, following World Health Organization guidance (World Health Organization, 2016), and discussions among the first two authors, the nature of the content of the policy documents was classified along the continuum of:

- **Gender blind**: content that ignores gender norms, roles and relations and differences in opportunities and resource allocation for women and men.
- **Gender sensitive**: content that indicates awareness of the impact of gender norms, roles and relations, but no remedial actions are developed.
- **Gender specific**: content goes beyond indicating how gender may hinder health of adolescents to highlighting remedial measures, such as programmes for adolescent girls and/or boys.
- **Gender transformative**: content that includes ways to transform harmful gender norms, roles and relations.

Building on this, the discourse analysis draws on the work of Bacchi (Bacchi and Eveline, 2010; Bacchi, 2016) and the ‘What’s the Problem Represented to be?’(WPR) approach. This approach asks questions related to what is problematized and what is left unproblematized in policies, i.e. what is missing, as well as what assumptions are made and how these have come about. By elucidating what effects are produced by this representation and how it has been (re)produced, it identifies opportunities for disruption. It is, therefore, a useful approach in analysing both the dominant and marginalized discourses as well as the ‘silences’ related to gender in South African adolescent health policy documents. The first author led the analysis of the relationship between the discourses, including those that were dominant and marginalized. The ‘silences’, i.e. what was missing in relation to gender and adolescent health, were measured against the global and national literature, as well as subject and contextual knowledge. The initial analyses were reviewed by the second and third authors, and consensus was achieved through discussions and further refinement of the analysis.

### Positionality and reflexivity

Doing a critical discourse analysis of adolescent health policy documents is a process of interpretative policy research. It requires us to be aware of our positionality in relation to the research and to our own epistemological views of the world. This positionality includes a situatedness as feminist researchers. In addition, the lead author has more than 20 years’ experience of working in gender, HIV and health programmes in South Africa.

## Findings

In this section, we first present the content analysis of policy documents, followed by our critical discourse analysis.

### Description of content of policy documents

Within the specified time range and scope of research, we found 15 government policy documents that are relevant to adolescent health, with different lead departments, actors and diverse foci, as presented in Table S1.

Among these 15 policies, six list the National Department of Health (NDoH) as the formal lead actor, one lists both the Department of Basic Education (DBE) and the NDoH as co-leads and two list DBE as the lead actor. The balance lists a range of multiple actors, including the Presidency, the Department of Social Development, the South African National AIDS Council, the Department of Justice and Constitutional Development, as leads.

In terms of focus and specificity, only one policy has a focused mandate on adolescents, namely, the Adolescent Sexual Reproductive Health Rights (ASRHR) Framework Strategy. This is also the only one to follow the WHO in delineating adolescents as 10–19 years, distinguishing between early, mid and late adolescence. Two policies focus on adolescents with youth (with inconsistent age ranges), the National Adolescent and Youth Health Policy (AYHP) and National Youth Policy, while five consider them with children. The remaining seven policy documents cover topics important for adolescents but are directed to the general population.

Many of the policy documents focus largely on key SRHR issues and HIV/AIDS, or on other issues such as mental health and nutrition in separate vertical policies and programmes. Only one policy, the National Adolescent Youth Health Policy, is more comprehensive. Figure S1 illustrates that most policies emerged after 2010, and this mirrors the global and national context at the time, that of the Millennium Development Goals leading into the SDG era, as well as the prioritization of HIV in South Africa.

The analysis of the content of the national government policy documents highlights that key gender concepts such as ‘sex’, ‘gender’, ‘gender in/equality’, ‘gender identity’, ‘sexual orientation’, and the like are defined in only three documents (Table S1). Even if gender and/or gender inequality are listed as social and contextual factors that impact on health, and/or are defined in the glossary of terms, there are no detailed gender analyses that are a systematic analysis and response to gender inequality as a social and structural determinant of power relations at individual, institutional and systems levels.

As shown in Table S1, in instances in which gender is addressed in policy documents, most documents are gender sensitive (i.e. they have content that indicates some awareness of the impact of gender norms, roles and relations but no remedial actions), with some having gender-specific responses overwhelmingly for adolescent girls and young women. Adolescent boys and young men are only briefly mentioned in three policies. Only two of the policies could possibly be classified as gender transformative, with some mention of intention or suggested interventions that transform harmful gender norms, roles and relations, but with little detail provided.

Despite there being some acknowledgement that adolescents face many contextual challenges and are not homogenous, the vast majority of the policy documents explicitly and implicitly refer to adolescents as largely homogenous and do not reflect their diversity, either in terms of identities or social contexts. Across the policy documents, there is a range of descriptions of historical and present social and structural determinants. This often lists, consecutively, poverty, violence and inequalities based on race, class, gender, ability, geographic location and sexual orientation and gender identity, as seemingly separate determinants, without addressing the intersectionality of these.

All the policy documents make reference to the South African Constitution and legal and policy frameworks that centre human rights. However, only three of the policies include make explicit reference to the rights and empowerment of adolescents. Despite there being some consultation with learners during the drafting of the National Policy on the Prevention and Management of Learner Pregnancy at School, only the AYHP (2017) explicitly describes the engagement of adolescents, as a key actor group, in the policy development process.

### Dominant and marginalized interrelated discourses and ‘silences’

Building on the content analysis that describes what is included in the policy documents at a literal level, through critical discourse analysis we deepen our understanding of what is being said in more indirect ways. Importantly, the content of policy documents is evidence of the ideas, understandings and assumptions of multiple policy actors in the South African context and as such provides some insights into in how and why gender is understood by policy actors in policies relevant to adolescent health.

This analysis reveals through a gendered lens some of the key interrelated, often juxtaposed, dominant and marginalized discourses, as well as the ‘silences’, embedded in the policy documents. We explore the discourses that shape how gender is conceptualized and the influences this has on how gender is addressed by these policy documents.

### Gender as biological sex and a fixed category vs social processes

Dominant discourses throughout South African adolescent health policy documents construct gender as a fixed category rather than as social processes, and this is expressed in various ways. It is found in the ways in which gender is equated with biological sex, without recognizing the social construction of femininities and masculinities, as well as the relational aspects of gender. As a result, there are conceptual conflations, e.g. assuming gender to mean a focus on girls, separate from the social contexts, relations, structures and actors that create inequality. Further, as mentioned earlier, the representation of adolescent boys, both in terms of their own health (which is also shaped by gender) and in terms of their role in pregnancy, e.g. and gendered power relations, is conspicuously absent.

A further set of interrelated dominant discourses, explicitly and implicitly, emphasizes fixed binaries, such as ‘boys/girls’, ‘masculine/feminine’ and ‘heterosexual/homosexual’. Discourses representing key gender concepts, including sexual orientation and gender identity, as fluid, diverse and non-binary, are more marginalized and less visible in the policy documents. These binary discourses are related to the understandings and assumptions of gender as a fixed individual characteristic, as discussed above. Together they reproduce dominant ways of conceptualizing gender well as how they construct the subjects of those policy solutions, making invisible non-binary persons and also inhibiting understandings of a continuum of diverse, dynamic and nuanced forms of sexual orientation and gender identity for adolescents, as well as among the general population. Correspondingly, many of the policy documents tended to catalogue groups as mutually exclusive target populations, including LGBTIQA+ persons, adolescent girls and young women, particularly in relation to HIV infection and sexual and gender-based violence, ignoring cross-cutting determinants and overlapping identities, as illustrated by the example below:

*Goal 3: Reach all key and vulnerable populations with customized and targeted interventions for*:

- *Vulnerable populations for HIV and STIs*
- *Adolescent girls and young women*
- *Children including orphans and vulnerable children*
- *People living in informal settlements*
- *Mobile populations*
- *Migrants and undocumented foreigners*
- *People with disabilities*
- *Other lesbian, gay, bisexual, transgender and intersex (LGBTI) populations*

(Source: NSP on HIV and STIs and TB (2016−2020))

Discourses representing how gender inequality intersects and compounds other axes of inequality such as race, class and (dis)ability, e.g. in the South African context, are ‘silent’ in the analysed policies. If adolescent health is not located in these intersecting and compounding inequalities and power relations, they will continue to be seen as separate and hence insufficiently addressed or problematized by the policy documents.

### Corresponding focuses on vulnerability and risks vs rights

The categorical focus on adolescent girls and young women is problematic in the way it shapes how gender is addressed. It constructs them as inevitably vulnerable, often without an acknowledgement of the multiple power relations that generate intersecting and compounding inequalities; therefore, their health is constructed out of context of power relations and as apolitical. By consistently and uncritically representing adolescent girls and young women and other categories as vulnerable, the idea that this vulnerability is expected, unchangeable or even ‘normal’ is supported.

While co-existing with the categorical focus on ‘addressing gender as addressing girls’ described above, most of the policy documents construct adolescent health and sexuality negatively and predominantly about risks and problems. This is juxtaposed against more positive understandings of health and well-being and comprehensive constructions of adolescent health in recent national and global policy documents. However, the majority of the policy documents framed adolescent sexual and reproductive health outcomes as a result of engaging in ‘risky behaviour’, e.g. having sex too early, having too many sexual partners, not making the use of contraception and contracting HIV. Not only are girls and femininities generally constructed as being more ‘at risk’ or ‘vulnerable,’ without any critique of why and how this has come about, but they are also constructed as the target groups of policy measures with no attempt made to address the power relations, systems, and actors that construct that vulnerability. Across the policy document, *‘*silences’ also include attention to boys in terms of their own health and through a gendered lens, as well as LGBTIQ+ adolescents beyond issues pertaining to HIV.

Juxtaposed against these dominant discourses of ‘risks’, described above, we also identified marginalized discourses representing adolescent rights. These representations see adolescents from a broader rights approach as opposed to a more public health approach. It views adolescents as societal assets whose contributions can be nurtured and amplified through meaningful engagement and participation, including outside of the health sector. These kinds of discourses are only represented in recent policies, and mostly in relation to SRHR, as illustrated in the example below.

*Investing in the sexual and reproductive health of adolescents and youth is of a great imperative. Through the advancement of sexual and reproductive health and rights for adolescents acknowledging and including those underserved groups such as lesbians, gay, bisexual, transgender and intersex(LGBTI), sex workers, HIV positive youth and those living with a disability calls for the development of an inclusive agenda that intends to promote the quality of life and the right to choose whether and when to have children; the right to exercise sexuality free of violence and coercion; the right to seek pleasure with respect for other people’s rights; the right to protect fertility; and the right to access modern techniques for the prevention, diagnosis and treatment of sexually transmitted infections.* (Source: ASRHR Framework Strategy (2015−2019)).

While there is a recognition of a rights perspective within the focus on sexual and reproductive health, there is relative ‘silence’ across the policy documents with regard to the inclusion of interventions and programmes that address the full spectrum of rights. This includes sexual rights, particularly in ensuring comprehensive services (one being access to abortion) and how these are related to gender equality, human rights and social and reproductive justice.

More positive constructions of sexuality are emerging, but also contested, in recent policies which include Comprehensive Sexuality Education (CSE). There is much public debate linked to sexuality education and strong resistance from some actors, such as parents and religious groups, to the plans of the DBE to implement a new CSE curriculum aligned to global best practices. The multiplicity of discourses, some emphasizing risk and vulnerability and some articulating rights even if challenged, reflects how contested social realities and interests permeate into the content of policy documents. This dynamic between discourses of risks vs rights described above also highlights the underlying assumptions and beliefs around adolescence, health and adolescent sexuality held by a range of policy actors. Importantly, the actors shaped how the ‘problem’ and ‘solutions’ as represented in these policies, which has implications both in terms of what this means conceptually, but also how services are planned and implemented.

### Policy implications: addressing ‘symptoms’ or transforming gender power relations

With the above in mind, we identified a general tendency across the policies where ‘symptoms,’ of gender power relations (such as gender-based violence) were foregrounded while excluding the underlying causal determinants. As an example of this, the extract below directs attention to violence as a concern for adolescent health and identifies post-violence care for meeting the immediate needs of survivors. This is an essential short-term response crucial in a country with extremely high prevalence rates of both HIV and GBV and where gender inequalities fuel and exacerbate the vulnerability of girls and women to both epidemics.

The policy documents do not, however, adequately acknowledge that violence in South Africa is gendered and shaped by intersecting and compounding past and present social and structural determinants, such apartheid and patriarchy, for example.

*Violence and substance abuse have major negative impacts on the health of adolescents and youth in South Africa and increase risks to physical and mental health and wellbeing. The abuse of drugs and alcohol is increasing among adolescents and youth, with alcohol abuse in particular linked to high levels of violence and motor vehicle accidents. Post-violence care is part of the comprehensive package of sexual and reproductive health and emergency services, but the provision of post-exposure prophylaxis for rape survivors remains inadequate.* (Source: National Adolescent Youth Health Policy (2017))

The underlying understandings across most of the policy documents are that adolescent health can be addressed using targeted, treatment-focused strategies rather than simultaneously including prevention-focused gender-transformative strategies that foreground gender and intersecting power relations. Across the policy documents there was almost never an attempt to dismantle or critique the social and structural determinants which lead to these ‘symptoms’ and instead the focus was placed on interventions to manage them.

Further, there was much ‘silence’ on how to transform gender inequality beyond the focus on providing services and biomedical and behavioural interventions for adolescent girls and young women. The effects of this are that these ‘symptoms’ are then addressed by providing services and interventions targeted largely at the individual level and responding to the immediate consequences. Importantly, while it is essential to address the short-term consequences of gender inequality, i.e. the ‘symptoms’, the longer term and cross-cutting determinants also need to be included in policy documents. Given the significant impact of both GBV and HIV, it is crucial to prioritize services. However, without the equal prioritization of underlying common determinants such as gender inequality, our responses will be partial and maintain − not transform − the status quo. While a service delivery focus on adolescent girls is an important practical component, the challenge remains how also to address and transform gender inequality strategically.

This decontextualized focus on ‘symptoms’ further reproduces understandings that adolescent health is largely the domain of the health sector and is mostly about providing services for health problems This deflects from the understandings that health is political and constructed by the unequal social, political and economic systems that are gendered and also require action by other sectors and actors.

Correspondingly, we found that policy documents predominantly focus on gender as an issue of importance at the micro-level (individual or interpersonal) and understandings of the role of gender inequality at the meso- (organizational) and macro- (structural) levels, are largely absent. Understanding gender as mainly about categorical thinking, counting girls vs boys in terms of sex-disaggregated data or gender parity, discounts the ability to see gendered processes that affect health and society as systems of power, including at the meso- and macro-levels that require transformation.

## Discussion

The landscape of policy documents relevant to adolescent health in South Africa consists of multiple actors and focuses and lacks coherence and alignment. Furthermore, the policy documents define and consider adolescents and their health with varying specificity. This highlights the tension between having policies that are adolescent-specific or having an Adolescent Health in All Policies approach (World Health Organization, 2017). While the Departments of Health and Education lead many policies, the plethora of government agencies involved flags the need for further multi-sectoral collaboration and coordination on adolescent health.

Within this fragmented landscape, the gender analysis of the content of South African adolescent health policy documents revealed that while gender is sometimes mentioned as a social determinant in some policy documents, little systematic gender analyses and gender integration are undertaken. The policy documents can be categorized as mostly gender sensitive (recognizing gender but not addressing it), at times gender specific (addressing girls needs pragmatically), but rarely gender transformative, i.e. in ways that change gender power relations. Intersectional approaches to understanding adolescent health were not present. While rights in the South African Constitution were always referenced, policy documents did not prioritize adolescent empowerment, with only one engaging adolescents in its development.

Our discourse analysis reveals that the superficial and non-transformative ways in which gender is addressed reflect its framing as an individual characteristic lost within the fragmented landscape of adolescent health policies in South Africa. Further, our analysis highlights the influence of multiple actors, as the content of the policies reflects and reproduces the range of understandings and discourses related to adolescence, adolescent health, gender and rights. These multiple discourses in the content of the policy documents provide insight into the complexity of perspectives of policy authors and actors, in terms of adolescent health and gender.

In contrast, the development of shared understandings of gender as socially constructed and the prioritization of gender inequality as key social and structural determinants of adolescent health would imply further policy coordination across sectors and corresponding policies. What is needed are broader conceptualizations of gender which open pathways for transformation and a disruption of power relations at the micro-, meso- and macro-levels of the health and broader systems.

Importantly, the content of policy documents is evidence of the understandings and the multiple ways in which gender and gender equality are represented in policy documents. It reflects the ideas, understandings and assumptions held by policy actors in the South African context and as such provide some insights into how and why gender is problematized in policies relevant to adolescent health. The findings contribute to the literature on how policies are socially constructed, influenced by the dynamic interactions between context, actors and processes located in organizational, national and global contexts (Ingram *et al.*, 2007; Weible *et al.*, 2012; Walt and Gilson, 2014).

Our analysis recognizes that the discourses described are interwoven with each other and are therefore interdiscursive and intertextual, and, interrelated to each other within and across the policy documents. Collectively, they produce and reproduce what is problematized and what is left unproblematized in policy documents, both how gender is conceptualized and the implications of this conceptualization for how gender is addressed in policies with meaningful consequences for the lives of adolescents.

Across the policy documents dominant discourses construct gender as equating biological sex and gender identity and sexual orientation as binary and heteronormative. A resonating thread of dominant discourses construct gender as equating girls and a focus on health problems which disproportionately affect women in ways which are decontextualized from power relations and the social and structural determinants that shape them. In addition, our findings illustrate that in most policy documents addressing gender is equated to addressing vulnerability and ‘risks’ and thus it responds only to immediate ‘symptoms’ of gender inequality and does not transform the upstream social and structural determinants and root causes such as the underlying patriarchal belief and practices that perpetuate gender power relations.

These dominant gender discourses co-exist and are juxtaposed against marginalized discourses related to adolescent health and gender that focus on rights, social and structural determinants of health, as exemplified through attention to CSE. We also identified certain ‘silences’ interwoven with the dominant and marginalized discourses. These ‘silences’ include gender as socially constructed, gender identities as fluid and non-binary, lack of attention to boys in terms of their own health and also in terms of gender power relations as well as LGBTIQ+ adolescents beyond issues pertaining to HIV.

These ‘silences’ are interrelated with marginalized discourses and embedded in the broader discourse of adolescent health, gender equality, human rights and social and reproductive justice, all of which is informed by the South African social and political context.

Collectively, these dominant, marginalized discourses and ‘silences’ produce and reproduce what is problematized and what is left unproblematized in policy documents, for they shape what is in health policy documents and what is excluded, with significant implications for policy subjects and implementation.

The constructions of gender and adolescent health and related discourses identified in our analysis produce and reproduce broader discourses present in South African society. These discourses are located in a history of structural violence under colonialism and apartheid and continuing neo-liberal economic policies, which collectively directly influence the health of all South Africans (Chopra and Sanders, 2004). This social and economic context is interwoven with and exacerbated by the legacies of structural inequalities, compounded by patriarchy and related constructions of masculinities and femininities. These have collectively resulted in South Africa’s being one of the most unequal societies, where gender inequality intersects and compounds other inequalities such as race, geographical location and class (Coovadia *et al.*, 2009; Hassim, 2014; Gouws, 2017).

Part of this historical and contemporary context has created interrelated dominant discourses related to adolescent health and sexuality, which are about social ‘ills’, disease and dangers of pregnancy, HIV and rape (Bhana *et al.*, 2019; Ngabaza and Shefer, 2019). Further, the HIV epidemic and dominance of HIV discourses also shape the dominant discourses as well as ‘silences’ in terms of what and who are focused on in adolescent health policy. Importantly, the discourses identified in our analysis are underpinned by many ideas and actor perspectives, related to gender and gender inequality in the South African context. Consequently, these discourses could also be playing a role in sustaining and reproducing complex patriarchal power relations. They raise the critical point that policy documents not only reflect the status quo but are also productive and, hence, potential foundations for transforming gender power relations. Future policies would benefit from foregrounding gendered power relations and the gendered social and cultural norms, which have an impact on adolescent health within the content of policy documents. The addressing and illustrating of these unequal power relations also need to be part of a comprehensive programmatic response to inequalities in adolescent health in South Africa.

### Implications for policies, programmes and systems

As shown in the findings, most policy documents outline programmes and interventions focused on adolescent girls and young women who bear the brunt of the dual epidemics of HIV and gender-based violence and are already the focus of many government and civil society services. In the South Africa context at the meso- and macro-levels, there are initiatives led by various actors such government and donors and at times complement and/or contradict the policy documents. These include the She Conquers campaign (Subedar *et al.*, 2018), the DREAMS initiative and other Global Fund programmes, which largely focus on HIV and adolescent girls and young women and on providing services.

While this service delivery lens is essential to ensure that their practical needs are met through gender-specific services and interventions to empower them, we would argue, at the same time, for a broader systems lens and the prioritizing of strategic gender-transformative programmes (George *et al.*, 2019a). This would include building on the growing body of evidence of gender-transformative approaches based on the foregrounding gender power relations, including the re-conceptualization of constructions of masculinities and femininities (Ellsberg *et al.*, 2018; Amin *et al.*, 2018). We therefore recommend that including gender-transformative responses and addressing gender inequality as a structural determinant should be integrated into both response and prevention to ensure a greater impact. Further, we recommend that these be implemented across sectors in order to disrupt and transform the power relations that create ill-health and, in so doing, re-politicize adolescent health and combine public health and rights-based approaches at micro-, meso- and macro-levels of systems.

As policy makers, implementers and researchers we also need to question how and why adolescent health becomes gendered in ways that largely reduce it to a focus on girls, problems, rather than a comprehensive focus on all adolescents and also on their well-being. We need to critically analyse how the social and political context mediates this and ‘silences out’ more positive discourses on adolescence health, in order to develop more adolescent-responsive and gender-transformative health policies and systems. While understanding that the main purpose of policies is to respond to problems and to address priorities, we need to consider a more comprehensive and gender-transformative perspective on adolescent health, as encouraged by the WHO (2017). In addition, in terms of the health system, we endorse the need for the development of a shared vision for adolescent health in South Africa, for a greater alignment of policies within and across departments as well as clear guidelines as to how the complementary multi-sectoral programmes should be implemented (World Health Organization, 2017; Cluver *et al.*, 2018; 2019; Toska *et al.*, 2019). For example, the COVID-19 pandemic has foregrounded and exacerbated the pre-existing and widespread scourge of GBV in South Africa − also highlighted through civil society activism and high-profile media cases. The newly launched National Strategic Plan on GBV and Femicide (2020–2030) and the attention given to it by President Ramaphosa and other actors is encouraging, although a huge gap still remains between these commitments and the reality on the ground in terms of gender and intersecting inequalities.

In order to realize a more comprehensive and cohesive response, we suggest there should be strong leadership at multiple levels, collaborative governance frameworks and the capacity to develop and implement policy as well as capacity to analyse and integrate gender into programmes beyond tick-box exercises. As illustrated in the findings, the dominant, marginalized and even ‘silent’ discourses have implications for how adolescent health is addressed at policy and programme level and how adolescents in their diversity experience the health system. It also raises the critical question as to the role actors in constructing the content of policy documents and what this means for reproducing or transforming gender power relations. We need to reconsider how the content of policies shapes and limits the programmes we implement and the pathways and processes for transformation within health and social systems in order to meet the needs of all adolescents and leave no one behind.

### Implications for gender integration

Our findings contribute to the literature on minimal gender integration and prioritization in policy documents, similar to the analyses of PMTCT policies (Nyamhanga *et al.*, 2017), lay health worker policy (Klugman, 2000; Daniels *et al.*, 2012) as well as global health policy (Gibbs *et al.*, 2012; Olinyk *et al.*, 2014). Further, our analysis of adolescent health policy documents contributes to the body of evidence highlighting the absence of in-depth gender analysis as part of transforming inequitable systems and structures within the health system (Theobald *et al.*, 2017; Witter *et al.*, 2017; Morgan *et al.*, 2018; JHPIEGO, 2016). In terms of policy processes this could be due to the absence of gender expertise and/or a gender champion as well as structural issues that influence whether it reaches the policy agenda and actual policy, as described by Daniels *et al*. (2012) and Klugman (1999). The analysis of the content of policy documents highlights multiple gendered discourses that provide insight into the complexity of perspectives of policy authors, thus adding to the literature on how policies are socially constructed processes (Klugman, 2000; Mannell, 2014; Koduah *et al.*, 2015; Lombardo *et al.*, 2017).

We acknowledge that policy documents may have limitations and boundaries in terms of space and scope and that these will shape what is included in the final policy document. While it may not be reasonable to expect in-depth, detailed gendered analyses within the policy documents themselves, an acknowledgement of power, context and the relationships between different issues affecting adolescent health is crucial if these important factors are to be integrated into programming. Further, there should be some caution around the way policy documents simplify certain issues for the sake of brevity, as it is also important to acknowledge some of the complexities if these issues are to be effectively and comprehensively addressed.

Our research also echoes the call by others for greater attention to addressing gender inequality as a social and structural determinant of adolescent health, as a key to transforming gender inequalities in health (Sen *et al.*, 2007; George and Amin, 2020). In addition, they also contribute to the debates and productive tensions in the gender mainstreaming literature, which calls for a focus on addressing unequal power relations and promoting a bigger contextual picture of how gender intersects with race, class, sexuality and nationality, beyond any quantitative gender parity approaches (Ravindran and Kelkar-Khambete, 2008; Garcia-Moreno and Amin, 2019).

### Implications for research

As a theoretical and analytical approach, combining content and critical discourse analyses has offered new lines of enquiry. Applying a critical discourse analysis to the content of policy documents reveals the underlying understandings, values and meaning making of actors, as central to the construction of policy (Parkhurst *et al.*, 2015; McDougall, 2016; Gilson *et al.*, 2018). Further, it has enabled critical reflections and deepened the analyses of the relationships between the content of adolescent health policy and social context in South Africa and opens up spaces for engagement with policy actors, building on other authors (Harmer, 2011; Parkhurst, 2012; Payne, 2014; Parkhurst *et al.*, 2015; Evans-Agnew *et al.*, 2016). Our application of Bacchi’s WPR approach also enabled critical engagement in terms of what is ‘problematized’ and also what is left out of adolescent health policies, as applied by others (Payne, 2014; Archibald, 2019; Baum *et al.*, 2019; Pringle, 2019). Our analysis also shows that in the policies, power and relational aspects of gender are not adequately analysed, and this underscores the importance of paying greater attention to power relations that construct individual health (e.g. that of adolescent girls) and asks the questions as to what social and structural systems create, thus reiterating the importance of the interrelationship between the personal and the political.

Further, our analysis also shows that in the policies, power and relational aspects of gender are not adequately analysed. We support the call by Morgan *et al*. (2018) for sex disaggregated data to be a trigger for further research and to deepen our analyses on gender power relations, including constructions of masculinities and femininities and what these mean for health and broader social systems. In addition, we also suggest further intersectional analyses in terms of gender and adolescent health i.e. how gender and other axes of power and marginalization intersect and compound each other. Understanding the perspectives, experiences and roles of policy actors, in terms of how gender inequalities are produced and reinforced in health policy processes, is an area for further research in South Africa.

## Limitations

The data analysed in this paper are policy documents that both provide an opportunity for in-depth analysis of their content but also has significant limitations. Viewing policy documents as products or ‘artefacts’ of the policymaking process allows us to explore how these texts represent ideologies and beliefs which are part of the social and political context. However, using policy documents alone has certain limitations in terms of understanding the dynamic interaction of other policy elements such as policy processes and actors, as the voices of the latter are not present in this paper. This will thus be the focus of a forthcoming research paper.

This paper shares insights from an in-depth gender analysis of 15 policy documents relevant to adolescent health in the South African context. It does not aim to provide empirical generalizations to other sectors or contexts, although the authors acknowledge how gendered policy approaches to education and employment policy, as structural determinants, impact on adolescent health.

## Conclusion

This assessment of South African adolescent health policy documents foregrounds how gender is not systematically incorporated across a fragmented policy landscape and that gender inequality and intersecting axes of inequality are not sufficiently analysed as social and structural determinants of adolescent health. Our findings show that how gender is conceptualized in policy documents, e.g. as equating biological sex and constructing adolescent health to be largely about vulnerable adolescent girls, rather than about social and structural power relations, has implications for gender-transformative multi-sectoral approaches.

Our analysis contributes to the understanding that policies are not just words or decontextualized texts. Their content is socially constructed and reflects and reproduces the conceptual understandings and ideological terrain related to gender, embedded in the policy documents. Our research makes visible the often taken for granted ‘problems’, ideas and interpretations, with implications for how they are addressed by means of ‘solutions’. We conclude that how gender is conceptualized matters, both for policy analysis and for praxis, and that policy documents can be foundations for transforming gender and intersecting power relations.

## Supplementary Material

czab041_Supp

## Data Availability

Data for this paper are government policy documents and no additional data were generated. These policy documents can be made available should that be required.
