# Supplementary material for: Policy foundations for transformation: a gender analysis of adolescent health policy documents in South Africa
Source: Health Policy Plan. 2021 Apr 14;36(5):684–94. doi: 10.1093/heapol/czab041 (PMC8248976; doi:10.1093/heapol/czab041)
Supplement: czab041_Supp [file czab041_supp.zip › Figure 1 Policy foundation _16 March 2021.docx]

**Figure 1. Timeline of policy documents**
