# Supplementary material for: Policy foundations for transformation: a gender analysis of adolescent health policy documents in South Africa
Source: Health Policy Plan. 2021 Apr 14;36(5):684–94. doi: 10.1093/heapol/czab041 (PMC8248976; doi:10.1093/heapol/czab041)
Supplement: czab041_Supp [file czab041_supp.zip › Table 1 policy foundations_ 16March 2021 .docx]

| **#** | **Policy & Mandate** | **Lead Actors** | **Definition and framing of Adolescence** | **Adolescent Health** | **Definitions and framing of Gender** | **Intersectionality** | **Rights and engagement** |
| --- | --- | --- | --- | --- | --- | --- | --- |
| 1 | Adolescent Sexual Reproductive Health Rights (ASRHR) Framework Strategy (2015)  Adolescent specific | DSD | - Age range: 10-19 years (early adolescence 10-14, late adolescence 15- 19) - Adolescence is defined in the policy as well as the overlap with youth - Adolescents framed as diverse; e.g. groups that do not have access to services include LGBTQI+ or HIV positive adolescents | - The Framework has a focus on SRHR for adolescents as a basic human right and outlines various priorities and components related to improving coordination and strengthening service delivery and creating community support networks for adolescents | - Gender and key gender concepts are defined, including sexuality and LGBTIQ+ - Gender is presented beyond a male/female binary and as fluid, diverse and socially constructed - Some analysis of gender and mention and acknowledgement of social determinant of SRHR - Gender-transformative interventions mentioned i.e. including ways to transform harmful gender norms, roles and relations noted in content. CSE is seen as an to address gender power relations and promote access to services for all adolescents | - Notes diverse and multiple forms of inequality particularly around access to education and services - No detailed intersectional analysis | - Aligned to Constitution and to global and national rights policies - Rights and agency, particularly related to access to SRHR services and non-discrimination, is a strong thread across the policy - Adolescents not mentioned as part of the development of the policy |
| 2 | National Adolescent and Youth Health Policy (AYHP) (2017)  Adolescents combined with youth, both in and out of school | NDoH | - Age range: 10-24 years - Adolescence is defined as a period of emotional and social development, growing independence and changing relationships within families, friendships and communities - Adolescents framed as largely a homogenous group | - Policy has broad positive definition of health with objectives related to the use of innovative technology, provision of SRHR services integrated with HIV, prevention of violence and substance abuse, promotion of healthy nutrition and empowerment to engage with policy and programming e.g. AYHFS | - Gender and key gender concepts are not defined - Gender is largely presented as a male/female binary and heteronormative ways. - No substantial gender analysis and some acknowledgement of gender as part of the social determinants of health - Gender is noted in a basic and implicit way (e.g. mentions that patriarchal gender norms can reduce freedom to seek and secure health services, - Gender-specific interventions i.e. highlighting remedial measures with a focus on AGYW as well as note of and MMC for boys. Gender-based transformative programmes in collaboration mentioned once with DSD and CBOs but minimal detailed | - Lists that social and structural deprivations such as poverty, income shocks, mental health distress, stigma, as 'intersecting’ with gendered norms that disempower girls and women, as key drivers of risky behaviours and poor health outcomes understood within a social ecological framework that recognises the interconnected influences of family, peers, community and society - No detailed intersectional analysis | - Aligned to Constitution and to global and national rights policies - Adolescent rights, agency and engagement is a central tenant of the policy - Adolescents explicitly mentioned as part of the development of the policy |
| 3 | Child and Adolescent Mental Health Policy Guidelines (2003)  Adolescents combined with children | NDoH | - Age range: prenatal period (conception to birth), childhood (birth to 9 years) and adolescence (12 to 18 years) - Adolescence not defined but policy mentions childhood and adolescence as developmental stages but does not give a detailed overview of adolescence - Adolescents framed as largely as homogenous, despite noting that different contexts shape mental health | - Policy has a broad understanding of child and adolescent mental health, addressing risk and protective factors across various domains such as individual, family school and community | - Gender and key gender concepts are not defined - Gender is largely presented as a male female binary and heteronormative ways - Gender is noted in an basic way (e.g. is noted to influence vulnerability but in a way that doesn’t question gender power relations - Gender-blind interventions noted in content i.e. ignores gender norms, roles and relations and differences in opportunities and resource allocation | - Describes social and contextual factors such a poverty, intellectual disabilities, physical, emotional and/or sexual abuse, experiencing or witnessing violence - No detailed intersectional analysis | - Aligned to Constitution and to global and national rights policies - Rights, agency and engagement of adolescents not mentioned - Adolescents not mentioned as part of the development of the policy |
| 4 | Integrated School Health Policy (2012)    Adolescents combined with children  Grades: Reception- 12 All learners including learners with special needs | ND0H  DBE | - Age range: None - Adolescence is defined as a separate developmental stage - Adolescents are framed as largely homogenous | - The focus of the policy is on the improvement of the general health of school-going children as well as the environmental conditions in schools - Adolescents are mentioned in the sections dealing with ARV adherence, mental health and risky behaviour as well as the importance of providing services in an adolescent friendly manner | - Gender and key gender concepts are not defined - Gender is largely presented as a male/female binary and in heteronormative ways - No substantive gender analysis and not acknowledged as social determinant e.g. no recognition of how issues such as violence are gendered. - Gender-specific interventions with a focus on girls, but notes boys to be provided with information on male circumcision | - Situational analysis of social inequality and access to basic services and how that impacts on children - No detailed intersectional analysis | - Aligned to Constitution and national and global policies - Rights, agency and engagement of adolescents not mentioned. - Adolescents not mentioned as part of the development of the policy, however participation of learners noted as essential to implementation through established leadership structures and clubs |
| 5 | National Policy on HIV, STIs and TB for learners, educators, school support staff and officials in primary and secondary schools in South Africa (2017)  Adolescents combined with children  Learners from Grade 1-12, educators and support staff | DBE | - Age range: None - Adolescence not defined - Adolescents are framed as largely as homogenous but policy notes that they have specific rights in terms of access to contraceptive and HIV testing and termination of pregnancy | - The focus of the policy is to improve coordination of the response to HIV, STIs, TB and unintended pregnancy, to accelerate implementation of a comprehensive strategy for prevention, treatment, care and support | - Gender and key gender concepts are defined - Gender is presented beyond male/female binary and as a fluid, diverse and socially constructed - Includes some gender analysis of vulnerability beyond just mentioning girls and young women, but also affecting boys - Gender-transformative interventions noted including ways to transform harmful gender norms, roles and relations e.g. The CSE curricula is seen as one of the main mechanisms to talks about gender sexual orientation and gender identity and power relations | - Social and structural drivers noted, inlcuding access to information and not be discriminated against, inlcuding in terms of access to CSE - No detailed intersectional analysis | - Aligned to Constitution and national and global policies. - Rights, agency and engagement of adolescents is mentioned in order to make informed life choices to protect themselves from HIV, STIs and TB as well as unintended pregnancy - Adolescents not mentioned as part of the development of the policy though some leaners were consulted as part of stakeholder groups |
| 6 | National Policy on the Prevention and Management pf Learner Pregnancy at School  (Draft 28 June 2018)  Adolescents combined with children  Learners from Grade 1-12 | DBE | - Age range: None - Adolescence is not defined as policy only makes reference to learners - Adolescents are (implicitly) framed as homogenous | - The focus of the policy is the prevention and management of learner pregnancy at school and through providing comprehensive SRH services, inlcuding CSE, HIV/AIDS services and access to termination of pregnancy, access to antenatal care and to ensure schools provide a stigma-free, non-discriminatory and non-judgemental environment for pregnant learners, pre- and post-delivery | - Gender and key gender concepts are not defined - Gender is largely presented as a male/ female binary and in heteronormative ways - No substantive gender analysis but some recognition as a social determinant in that female learners, are particularly vulnerable and often exposed to sexual and gender-based violence, sometimes leading to coercion and assault, including rape. - Gender specific interventions in terms of a focus on girls as part of the pregnancy policy mandate, but also gender transformative with a focus on CSE as essential | - No detailed intersectional analysis | - Aligned to Constitution and national and global policies - Rights, particularly the rights of girls to education mentioned - Adolescents not mentioned as part of the development of the policy although some learners participated in consultative workshops |
| 7 | National Policy Framework on Child Justice (amended in 2018)  Adolescents combined with children  Any person younger than 18 years in contact with the criminal justice system | DJ&CD | - Age range: None - Adolescence not defined and they are subsumed in the category with children - Adolescents are (implicitly) framed as homogenous | - The focus of this policy is on supporting any person younger than 18 years in contact with the criminal justice system - There is some recognition of mental health concerns and details of how this needs to be provided by both the Departments of Health and Social Development - Adolescent health and wellness is not addressed specifically | - Gender and key gender concepts are not defined - Gender is largely presented as a male/ female ways and in heteronormative ways - No substantive gender analysis and not acknowledged as a social determinant - Gender blind i.e. ignores gender norms, roles and relations but notes that girls and boys need to be housed separate | - No detailed intersectional analysis | - Aligned to the Constitution and global and national policies - Rights, agency and engagement of adolescents not mentioned - Adolescents not mentioned as part of the development of the policy |
| 8 | National Youth Policy (2015-2020)  Adolescents combined with youth | National Youth Development Agency | - Age range 14 to 35 years - Adolescence not defined - Young people are framed as diverse and non-homogenous and unequal but does not have a specific analysis and focus on adolescents | - The focus of the policy is the social and economic development of youth and has a section on health addressing HIV/AIDS prevalence, violence and substance abuse and notes that many of these behaviours start in adolescence - Notes that SRHR of youth should be supported by both schools and the family to enable access to necessary information and health care | - Gender and key gender concepts are not defined - Gender is largely presented as a male/ female ways and in heteronormative ways - No substantive gender analysis and minimal focus on gender as a social determinant - Gender sensitive with content which indicates awareness of the impact of gender norms, roles, and relations, but no gender specific interventions noted | - Mentions high level principles based on non-discrimination on the basis of age, gender, race, sexual orientation, disability or any other form of discrimination - No detailed intersectional analysis | - Aligned to Constitution and to global and national rights policies - Adolescent rights not mentioned specifically but the rights and participation of young people in society is noted as very important - Adolescents not mentioned as part of the development of the policy |
| 9 | National Development Plan (2012)  General population | Planning Commission | - Age group: None - Adolescence not defined - Young people are framed as being unequal and there is a commitment to them as priority for employment and access to education, but does not have a specific analysis and focus on adolescence | - The focus of this policy is on several areas of national development such as education, economic development etc. It has a section/chapter on health, but this is largely focussed on financing of health, National Health Insurance, HIV and issues around violence and safety | - Gender and key gender concepts are not defined - Gender is largely presented as a male/ female ways and in heteronormative ways - No substantive gender analysis and minimal focus on gender as a social determinant - Gender sensitive with content which indicates awareness of the impact of gender norms, roles, and relations, but no gender specific interventions noted | - Describes high level values related to gender equality and a commitment to addressing inequalities base on race and gender - No detailed intersectional analysis | - Aligned to Constitution and to global and national rights policies. - Adolescent rights not mentioned specifically but equal rights for all is mentioned - Adolescents not mentioned as part of the development of the policy |
| 10 | National Health Promotion Policy and Strategy (2015-2019)  General population | NDoH | - Age range: None - Adolescence not defined but policy recognises youth as a key target audience - Youth( Adolescents) are framed as largely a homogenous group | - The focus of the policy is on health promotion for the general population with emphasis on the process of enabling people to increase control over their health and its determinants, and thereby improve their health - Focus is risky sexual behaviour, of healthy lifestyle practices and healthy nutrition, physical activity to prevent obesity and abstinence from tobacco and alcohol as well as focus of SGVB prevention | - Gender and key gender concepts are not defined - Gender is largely presented as a male/ female binary and in heteronormative ways - No substantive gender analysis and not acknowledged as a social determinant - Gender specific interventions noted with a focus females, but also that males should be targeted in campaigns to increase levels of awareness towards of gender norms and broader involvement in health programmes | - Lists some key social determinants of health and notes that addressing the key social, behavioural and structural determinants of health and understood within a social ecological framework that recognises the interconnected influences of family, peers, community and society on health seeking behaviours - No detailed intersectional analysis | - Aligned to Constitution and to global and national rights policies - Rights, agency and engagement of adolescents not mentioned - Adolescents not mentioned as part of the development of the policy |
| 11 | National Strategic Plan for HIV, TB and STIs (2017-2022)  General population | SANAC | - Age range: 10-19 - Adolescence not defined - Adolescents are largely framed as homogenous but the policy notes that vulnerability varies by age, geography and gender | - The overall focus of this policy is on HIV, STI’s and TB for the general population. The policy includes prevention, adherence support, reaching all key and vulnerable populations, addressing the social and structural drivers, human rights and promotion of leadership and shared accountability - Adolescent girls and young women (AGYW) are in the category of key and most vulnerable populations | - Gender and key concepts are defined - Gender is largely presented as a male/female binary and in heteronormative ways, but LGBTIQ+ persons in programming - No substantive gender analysis and with minimal focus on gender as a social determinant e.g. survivors of GBV but not addressing structural issues such as patriarchy - Gender sensitive and gender specific interventions noted with a focus on AGYW and ABYW mentioned in terms of specific interventions e.g. Medical Male Circumcision. One mention of gender-transformative interventions but no detail | - Lists social structural factors such as poverty, inequality, inadequate access to quality education, poor nutrition, migration, gender inequality, gender-based violence, and alcohol and drug use as increase vulnerability to HIV, TB and STIs - No detailed intersectional analysis | - Aligned to Constitution and to global and national rights policies - Rights, agency and engagement of adolescents not mentioned but has Chapter focussed on Human Rights - Adolescents not mentioned as part of the development of the policy |
| 12 | Sexual and Reproductive Health and Rights: Fulfilling our Commitments 2011-2021 and beyond (2011)  General population | NDoH | - Age range: None - Adolescence not defined but policy recognises that SRHR services have had a focus on women of reproductive age and lists adolescent as part of several groups that have not received adequate attention and whose needs have not been met. - Adolescents are framed as largely a homogenous group | - The policy has a focus on SRHR for the general population and is grounded in key rights principles and outlines services that will be provided to all based on their diverse needs at the community level as well as the health systems components needed. | - Gender and key gender concepts are defined - Gender is presented beyond male/female binary and as fluid, diverse and socially constructed - Some gender analysis with a focus on SRHR and emphasis on gender equity and the barriers to access services e.g. poverty, gender-based violence etc - Gender-sensitive i.e. indicates awareness of the impact of gender norms, roles, and relations on SRHR services and supports CSE | - Acknowledges gender and other forms of inequity as key barriers to SRHR. - No detailed intersectional analysis | - Aligned to Constitution and to global and national rights policies. - Rights and adolescent engagement is implied through school and community and health service provision but not explicitly discussed or outlined - Adolescents not mentioned as part of the development of the policy |
| 13 | National Contraception and Fertility Planning Policy and Service Delivery Guidelines (2012)  General population | DOH | - Age range 12-19 years - Adolescence not defined - Adolescents are framed as not being homogenous in terms of contraceptive needs which may vary in terms of age, physical and emotional development, culture and maturity as well as life circumstances | The overall focus of the policy is on contraception for general population but has a dedicated chapter which focusses on the needs of specific and defined groups i.e. adolescents; LGBTI; men; sex workers, migrants and disabled persons. It outlines the legal context and access to contraception, HCT and termination of pregnancy and access to AYHFS | - Gender and some key concepts are defined - Gender is largely presented as a male/ female binary and heteronormative ways - No substantive gender analysis but acknowledgement that contraception has always been feminized and that men need to be included more as partners and for their own needs - Gender-sensitive i.e. indicates awareness of the impact of gender norms, roles, and relations and gender-specific interventions noted | - Notes that discrimination in terms of sexual orientation, sexual preferences gender, age and culture and access to education - No detailed intersectional analysis | - Aligned to Constitution and to global and national rights policies - Rights, agency and engagement of adolescents not mentioned specifically but part of general population - Adolescents not mentioned as part of the development of the policy |
| 14 | Strategy for the prevention and control of obesity in South Africa (2015-2020)  General population | NDOH but signed by Trade and industry, Education, Public Service and Administration | - Age range: None - Adolescence is not defined and they are grouped with children as policy only makes reference to adults and children - Adolescents are (implicitly) framed as homogenous | - The focus of the policy is on prevention and control of obesity of the general population and addresses nutrition, physical activity and links to NCDs, with no broader aspects of health e.g. mental health, SRHR etc. | - Gender and key gender concepts are not defined - Gender is largely presented as a male/ female binary and in heteronormative ways - No substantive gender analysis and not acknowledged as a social determinant - Gender is described to equate biological sex, despite some mention that gender inequality is interaction of complex social, cultural and bio- logical factors - Gender sensitive interventions noted | - Acknowledges that gender inequality is a complex interplay of social, cultural and biological factors and influence of age and race are mentioned - No detailed intersectional analysis | - Aligned to Constitution and to global and national rights policies - Rights, agency and engagement of adolescents not mentioned specifically but part of general population - Adolescents not mentioned as part of the development of the policy |
| 15 | National Policy Framework on the Management of Sexual Offences (2012)  General population | DJCS | - Age range: None - Adolescence not defined and they are subsumed in categories of vulnerable groups i.e.. women and children. - Adolescents are (implicitly) framed as homogenous | - The focus of this policy is on the prevention of secondary victimisation of victims of sexual offences and outlines key principles and procedures to address these. In the general population, with emphasis on women and children, without specifying adolescents | - Gender and key gender concepts are not defined. - Gender is largely presented as a male/female and in heteronormative ways - No substantive gender analysis and not acknowledged as social determinant but notes that women and children are victims vulnerable and victims - Gender inequality is noted in an implicit way e.g. to influence vulnerability does not question gender power relations - Gender sensitive with content that indicates awareness of the impact of gender norms and notes gender specific interventions | - Mentions equal and equitable access to services and prevention discrimination on access of race, class, a gender, disability and sexual orientation - No detailed intersectional analysis | - Aligned to the Constitution and global and national policies. - Rights, agency and engagement of adolescents not mentioned , but policy adopts a victim centred rights approach - Adolescents not mentioned as part of the development of the policy |
